# Ninety-degree angled collimator: a dosimetric study related to dynamic intensity-modulated radiotherapy in patients with endometrial carcinoma

**DOI:** 10.1186/s12885-023-11033-8

**Published:** 2023-06-06

**Authors:** Alparslan Serarslan, Yalçın Daştan, Telat Aksu, Rana Elif Yıldız, Bilge Gürsel, Deniz Meydan, Nilgün Özbek Okumuş

**Affiliations:** Department of Radiation Oncology, Faculty of Medicine, Ondokuz Mayıs University, Samsun, 55139 Turkey

**Keywords:** Dosimetry, Endometrial carcinoma, Intensity-modulated radiotherapy, Volumetric modulated arc therapy

## Abstract

**Background:**

Our purpose was to ensure that the dose constraints of the organs at risk (OARs) were not exceeded while increasing the prescription dose to the planning target volume (PTV) from 45 to 50.4 Gray (Gy) with the dynamic intensity-modulated radiotherapy (IMRT) technique. While trying for this purpose, a new dynamic IMRT technique named 90° angled collimated dynamic IMRT (A-IMRT) planning was developed by us.

**Methods:**

This study was based on the computed tomography data sets of 20 patients with postoperatively diagnosed International Federation of Gynecology and Obstetrics stage 2 endometrial carcinoma. For each patient, conventional dynamic IMRT (C-IMRT, collimator angle of 0° at all gantry angles), A-IMRT (collimator angle of 90° at gantry angles of 110°, 180°, 215°, and 285°), and volumetric modulated arc therapy (VMAT) were planned. Planning techniques were compared with parameters used to evaluate PTV and OARs via dose-volume-histogram analysis using the paired two-tailed Wilcoxon’s signed-rank test; *p* < 0.05 was considered indicative of statistical significance.

**Results:**

All plans achieved adequate dose coverage for PTV. Although the technique with the lowest mean conformality index was A-IMRT (0.76 ± 0.05) compared to both C-IMRT (0.79 ± 0.04, p = 0.000) and VMAT (0.83 ± 0.03, p = 0.000), it protected the OARs especially the bladder (V45 = 32.84 ± 2.03 vs. 44.21 ± 6.67, p = 0.000), rectum (V30 = 56.18 ± 2.05 vs. 73.80 ± 4.75, p = 0.000) and both femoral heads (V30 for right = 12.19 ± 1.34 vs. 21.42 ± 4.03, p = 0.000 and V30 for left = 12.58 ± 1.48 vs. 21.35 ± 4.16, p = 0.000) better than C-IMRT. While the dose constraints of the bladder, rectum and bilateral femoral heads were not exceeded in any patient with A-IMRT or VMAT, they were exceeded in 19 (95%), 20 (100%) and 20 (100%) patients with C-IMRT, respectively.

**Conclusions:**

OARs are better protected when external beam radiotherapy is applied to the pelvis at a dose of 50.4 Gy by turning the collimator angle to 90° at some gantry angles with the dynamic IMRT technique in the absence of VMAT.

## Background

Endometrial carcinoma is the most common gynecological malignancy worldwide, and the primary treatment is surgery. Adjuvant treatment is usually delivered with systemic therapy and/or tumor-directed radiotherapy (RT) according to age, previous treatment history, and/or prognostic risk group. RT can be delivered as vaginal brachytherapy and/or external beam RT (EBRT) [1–3].

An EBRT technique, intensity-modulated RT (IMRT), optimally assigns nonuniform intensities to tiny subdivisions of beams, which are known as rays or “beamlets.” The ability to optimally manipulate the intensities of individual rays within each beam permits greatly increased control over the radiation fluence, thereby enabling the custom design of optimal dose distributions. Additionally, a sharper falloff dose is achieved at the planning target volume (PTV) edge [4]. The high conformality achieved enables lower doses of ionizing radiation to be delivered to organs at risk (OARs), enabling the delivery of higher doses of ionizing radiation to the PTV. These properties result in increased cancer control and an improved toxicity profile [5]. IMRT has been accepted as the standard EBRT technique in patients with endometrial carcinoma, based on the results of phase II [6] and phase III [7] studies performed in the context of theoretical knowledge obtained from dosimetric and retrospective studies [8–10].

IMRT can be applied using fixed-gantry techniques, such as dynamic IMRT, or rotational techniques, such as volumetric modulated arc therapy (VMAT). In dynamic IMRT, the optimized fluence distribution is delivered using the movement of each multileaf collimator leaf during irradiation [4]. VMAT is an advanced IMRT technique developed to achieve high conformality, which allows dynamic modulation of dose rate, gantry rotation speed, and multileaf collimator shaping during irradiation [11]. The radiation oncology department of our university hospital began applying dynamic IMRT with a Clinac DHX (Varian Medical Systems, Palo Alto, CA, USA) in 2011 and VMAT with Truebeam (Varian Medical Systems, Palo Alto, CA, USA) in 2017 for endometrial carcinoma. The recommended adjuvant EBRT dose prescription for microscopic disease is between 45 and 50.4 Gy in patients with endometrial carcinoma [2]. An increased dose of ionizing radiation provides better results in cancer treatment [12]. Unfortunately, the prescribed dose could not be increased from 45 to 50.4 Gy because the resulting dose exceeds the OAR dose constraints in dynamic IMRT. While attempting to further reduce the OAR doses during dynamic IMRT treatment planning for endometrial carcinoma patients, we realized that we could achieve better protection of OARs when the collimator was angled to 90° at some gantry angles. Here, we report this technique because not all RT centers have the financial resources for VMAT, particularly in developing countries.

In this article, we describe a new dynamic IMRT technique for patients with endometrial carcinoma. This new irradiation technique, named 90° angled collimated dynamic IMRT (angled IMRT = A-IMRT) planning, was dosimetrically compared to both the 0° angled collimated dynamic IMRT (conventional IMRT = C-IMRT) and VMAT planning techniques.

## Methods

### Ethics statement

This study was performed in accordance with the Declaration of Helsinki and approved by the local ethics committee of the Faculty of Medicine of Ondokuz Mayıs University, Samsun, Türkiye (application number: 2021000285-2; acceptance date: 18/6/2021 and acceptance number: 2021/285). All patients provided written informed consent prior to participation in the study.

### Patients

Twenty patients with endometrial carcinoma who underwent a total abdominal hysterectomy, bilateral salpingo-oophorectomy, and pelvic-paraaortic lymphadenectomy were included in this comparative planning study. All patients had stage II disease according to the International Federation of Gynecology and Obstetrics surgical staging system for endometrial cancers.

In our study, the number of patients was determined according to the power analysis performed with the values obtained from the study of Deng et al. The power of Deng et al.‘s study was found to be 100% according to the D2 [conformal radiotherapy (CRT) = 4650.8 ± 48.9, IMRT = 4907.0 ± 47.9, VMAT = 4962.2 ± 22.5; and pairwise statistical difference: CRT vs. IMRT, p < 0.001; CRT vs. VMAT, p < 0.001; IMRT vs. VMAT, p = 0.002] and alpha (= 0.05) values with the number of patients (n = 15) obtained from the same study [13]. After this determination, we planned our study with 20 patients in order to strengthen our study even more.

### Simulation

EBRT planning was performed three-dimensionally using a computed tomography (CT) simulator (Aquilion LB; Toshiba Medical Systems, Otawara, Japan). Patients were immobilized in the supine position with both arms raised above the head. CT imaging was performed twice for each patient, with and without intravenous contrast material, with a slice thickness of 3 mm, with a comfortably full bladder and empty rectum, and under free breathing. The data sets were transferred to a treatment planning system (TPS) (Eclipse 13.7.16; Varian Medical Systems) through a digital imaging and communications in medicine (DICOM) network.

### Definition and contour of targets

All contouring was conducted by one gynecological radiation oncologist. The clinical target volume (CTV), PTV, and OARs were defined using individual axial CT slices. The CTV was contoured in accordance with the NRG Oncology/Radiation Therapy Oncology Group (RTOG) Consensus Guidelines [14] and the Target Volume Delineation and Field Setup guidance [15]. CTV-1 included the vaginal cuff. CTV-2 included paravaginal/parametrial tissues without the vaginal cuff. CTV-3 included the common iliac, external iliac, internal iliac, and presacral nodal regions. Bone and muscles were removed from CTV. PTV-1, PTV-2, and PTV-3 were defined as additional 15 mm, 10 mm, and 7 mm uniform margins in all directions around CTV-1, CTV-2, and CTV-3, respectively. PTV-total consisted of PTV-1, PTV-2, and PTV-3 [15].

### Definition and contour of organs at risk and normal tissue

OARs included the bone marrow, bladder, rectum, bowel, and femoral heads [15]. Bone marrow was contoured from the L4 vertebral body to the ischial tuberosities, including L4–5, pelvis, and sacrum [16]. The bladder was contoured from its base to the dome. The rectum was contoured from the rectosigmoid junction to the anorectal junction. The small and large bowels were defined as all individual bowel loops, then contoured together as one structure (i.e., the “bowel”). The bowel was contoured 2 cm above the last slice of PTV to its lowest extent in the pelvis, and it included the whole peritoneal space. Femoral heads were contoured to the level of the ischial tuberosities. All tissue except the PTV in the treatment field was defined as normal tissue (NT).

### Radiotherapy planning

Contrast-enhanced CT was used to better visualize the vessels. Contrast and non-contrast CT scans were superimposed for planning. RT planning of all patients was performed using non-contrast CT [14]. Treatment planning was performed using Eclipse® TPS for delivery to a linear accelerator (Varian Truebeam SN-2934 version 2.7) equipped with a 120 Millennium multileaf collimator (central 20 cm of the field used leaves 0.5 cm wide, whereas the outer field used leaves 1 cm wide). For each patient, three plans were created with the C-IMRT, A-IMRT, and VMAT techniques.

Dynamic IMRT planning (C-IMRT and A-IMRT) was performed with seven noncoplanar fields using 6-MV photon beams for each patient. The isocenter was regarded as the midpoint of the PTVs. Similar gantry angles of 75°, 110°, 145°, 180°, 215°, 250°, and 285° were used in C-IMRT and A-IMRT. All collimator angles in C-IMRT were 0°. In A-IMRT, the collimator angle was 90° for gantry angles of 110°, 180°, 215°, and 285°; it was 0° for gantry angles of 75°, 145°, and 250° (Table 1). Photon dose calculation was performed using the anisotropic analytical algorithm. Heterogeneity corrections were switched on during all dose calculations. The maximum dose rate was set to 300 monitor units (MU)/min. The dose calculation grid was set to 2.5 mm. Because this was a dosimetric study, the gantry angles in both dynamic IMRT plannings were similar to allow comparison of different collimator angles.

**Table 1:** Relationship between gantry angles and collimator angles in C-IMRT and A-IMRT

Field number	1	2	3	4	5	6	7
Gantry angles in C-IMRT & A-IMRT	75°	110°	145°	180°	215°	250°	285°
Collimator angles in C-IMRT	0°	0°	0°	0°	0°	0°	0°
Collimator angles in A-IMRT	0°	90°	0°	90°	90°	0°	90°

Abbreviations: C-IMRT = 0° angled collimated dynamic IMRT planning technique; A-IMRT = 90° angled collimated dynamic IMRT planning technique

VMAT planning was performed with three full arcs using 6-MV photon beams for each patient. The isocenter was regarded as the midpoint of the PTVs. The first arc ran clockwise from 181° to 179° with a collimator angle of 30°, the second arc ran counterclockwise from 179° to 181° with a collimator angle of 330°, and the third arc ran clockwise from 181° to 179° with a collimator angle of 90°. The photon optimizer (version 13.7) algorithm was used to optimize leaf position, dose rate, and gantry speed. Photon dose was calculated using the anisotropic analytical algorithm. Heterogeneity corrections were switched on during all dose calculations. The maximum dose rate was set to 600 MU/min. The dose calculation grid was set to 2.5 mm.

**Fig. 1:**
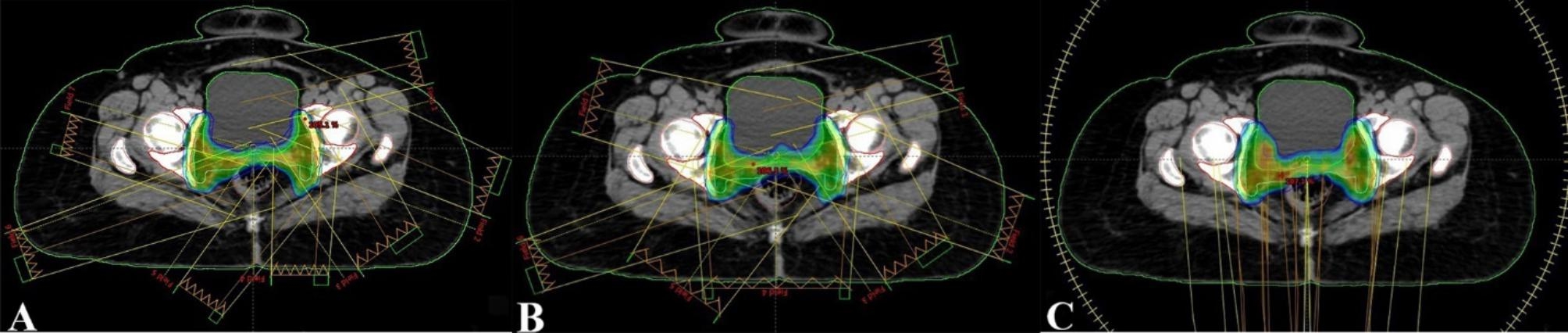
Axial computed tomography scan slice representations of a patient planned with C-IMRT (A), A-IMRT (B) and VMAT (C). Axial view of the 95% isodose of the prescribed dose in dose color wash, PTV (dark blue), CTV (cyan), bladder (green), rectum (brown) and femoral heads (pink) were shown

The planning objectives were identical for both dynamic IMRT and VMAT planning. The dose was prescribed to PTV, in accordance with the recommendations of the International Commission on Radiation Units and Measurements 83 report [17]. The prescribed dose was 50.4 Gy in all patients, delivered in daily fractions of 1.8 Gy. The dose was prescribed to cover 95% and 100% of the PTV and CTV, respectively. Care was taken to maintain a difference of < 10% between the prescribed and maximum doses. Axial CT scan slice representations of a patient planned with C-IMRT (A), A-IMRT (B) and VMAT (C) were shown in Fig. 1.

### Evaluation of RT planning

All treatment plans were evaluated according to the dose-volume histogram. The evaluated dosimetric parameters for PTVs were the volume of PTV receiving 95% of the prescribed dose (V95%), volume of PTV receiving > 107% but < 110% of the prescribed dose (V > 107%), dose received by 2% of the PTV (D2), dose received by 98% of the PTV (D98), dose received by 50% of the PTV (D50), conformity index (CI), and homogeneity index (HI). The total MUs of all treatment plans were also compared. For OARs, the evaluated dosimetric parameters were the volume receiving ≥ 40 Gy of the prescribed dose (V40) for bone marrow, volume receiving ≥ 45 Gy of the prescribed dose (V45) for the bladder, volume receiving ≥ 30 Gy of the prescribed dose (V30) for the rectum, volume receiving ≥ 35 Gy and ≥ 40 Gy of the prescribed dose (V35 and V40) for the bowel, and volume receiving ≥ 30 Gy of the prescribed dose (V30) for the femurs. Accepted dose constraints for OARs according to the literature are shown in Table 2 [6, 18, 19]. Because this was a dosimetric study, we did not compromise PTV coverage, even when OARs would be exposed to doses that exceeded the dose constraints.

**Table 2:** Dose constraints for organs at risk

Structure	Volume (%)	Target (Gy)
Bone marrow	< 37	≥ 40
Bladder	< 35	≥ 45
Rectum	< 60	≥ 30
Bowel	< 30	≥ 40
Bowel	< 35	≥ 35
Femoral heads	< 15	≥ 30

The HI was defined as: HI = (D2 − D98)/D50. The values of HI ranged between 0 and 1. The homogeneity increased as the HI value of PTV approached 0. The CI was defined as: CI = (TV_ref_/TV) × (TV_ref_/V_ref_), where TV_ref_ is the target volume (cm^3^) covered by the reference isodose, TV is the target volume (cm^3^), and V_ref_ is the volume (cm^3^) covered by the reference isodose. The values of CI ranged between 0 and 1. Conformality increased as the CI value of PTV approached 1. HI and CI were defined in accordance with International Commission on Radiation Units and Measurements 83 and 62 reports, respectively [20].

### Statistical analysis

Statistical analyses were performed using SPSS software (Version 22.0; SPSS Inc., Chicago, IL, USA). The values for all dosimetric parameters noted above for each treatment planning method were recorded and compared. The Friedman test was used for global p calculation to analyze the dosimetric differences between three planning techniques. The dosimetric differences between the two treatment plans were analyzed using the paired two-tailed Wilcoxon’s signed-rank test. Overdose rates of organs at risk were tested with more than two-group ratio test. In all analyses, *p* < 0.05 was considered indicative of statistical significance.

## Results

Dosimetric results obtained with dose-volume histograms of PTV, OARs, and NT were obtained from 60 treatment plans; three different plans were analyzed for each of the 20 patients. The volumes of the PTV and OARs in milliliters (ml) are shown in Table 3.

**Table 3:** The volumes of the planning target volume and organs at risk

Structure	Volume (ml)		
	Minimum	Maximum	Mean ± SD
PTV	896	1346	1093 ± 125
Bone marrow	738	1278	1059 ± 130
Bladder	147	1048	403 ± 197
Rectum	48	149	80 ± 29
Bowel	1107	4477	2452 ± 825
Right femoral head	81	175	126 ± 21
Left femoral head	87	166	128 ± 19

Abbreviations: PTV = planning target volume, SD = standard deviation

### Dosimetric parameters for planning target volumes

Although the desired 95% of the PTV received 100% of the prescribed dose in all plans, mean V95(%) values (*p* = 0.441) and mean D98 values (*p* = 0.737) were similar in A-IMRT and VMAT, respectively. Whereas the mean V > 107(%) (*p* = 1.0) and mean D2 (*p* = 0.335) values were similar in C-IMRT and VMAT, respectively, both mean V > 107(%) and mean D2 values were higher in A-IMRT than in C-IMRT (*p* = 0.000) or VMAT (*p* = 0.000) because of the developed maximum point doses. Homogeneity was better with C-IMRT than with VMAT (*p* = 0.04) or A-IMRT (*p* = 0.000); it was similar in both A-IMRT and VMAT (*p* = 0.255). Conformality was better with VMAT than with C-IMRT (*p* = 0.000) or A-IMRT (*p* = 0.000); it was better with C-IMRT than with A-IMRT (*p* = 0.000). Treatment was faster with VMAT (mean MU = 567 ± 68) than with C-IMRT (mean MU = 1036 ± 129; *p* = 0.000) or A-IMRT (mean MU = 1249 ± 149; *p* = 0.000); it was faster with C-IMRT than with A-IMRT (*p* = 0.000). The dosimetric parameters for PTV in each planning technique are shown in Table 4.

**Table 4:** Dosimetric parameters for planning target volumes in each technique

	Mean ± SD (minimum–maximum)			Global P
	**C-IMRT**	**A-IMRT**	**VMAT**	
V95 (%)	99.62 ± 0.28^**a**^ (98.62–99.91)	99.15 ± 0.43^**b**^ (98.24–99.81)	99.04 ± 0.70^**b**^ (97.77–99.94)	**0.002** ^*^
V > 107 (%)	0.02 ± 0.06^**a**^ (0.00–0.26)	0.20 ± 0.29^**b**^ (0.00–0.81)	0.02 ± 0.03^**a**^ (0.00–0.11)	**0.000** ^*^
D2 (cGy)	5288 ± 31^**a**^ (5201–5330)	5323 ± 52^**b**^ (5192–5436)	5277 ± 46^**a**^ (5216–5367)	**0.003** ^*^
D98 (cGy)	4948 ± 43^**a**^ (4828–5033)	4905 ± 42^**b**^ (4819–4962)	4898 ± 89^**b**^ (4752–5048)	**0.008** ^*^
D50 (cGy)	5208 ± 29^**a**^ (5120–5244)	5219 ± 28^**a**^ (5171–5266)	5176 ± 46^**b**^ (5112–5260)	**0.002** ^*^
HI	0.06 ± 0.00^**a**^ (0.05–0.08)	0.08 ± 0.01^**b**^ (0.05–0.12)	0.07 ± 0.02^**b**^ (0.04–0.11)	**0.006** ^*^
CI	0.79 ± 0.04^**a**^ (0.74–0.87)	0.76 ± 0.05^**b**^ (0.68–0.86)	0.83 ± 0.03^**c**^ (0.76–0.88)	**0.000** ^*^
MU	1036 ± 129^**a**^ (900–1297)	1249 ± 149^**b**^ (1048–1558)	567 ± 68^**c**^ (445–673)	**0.000** ^*^

Abbreviations: C-IMRT = 0° angled collimated dynamic IMRT planning technique; A-IMRT = 90° angled collimated dynamic IMRT planning technique; VMAT = volumetric modulated arc therapy; a,b or c = in the comparison between two groups, if the groups have the same letter, there is no statistically significant difference, but if the same letter is not found, there is a statistically significant difference; (*) means statistically significant = p < 0.05; V95(%) = volume of PTV receiving 95% of the prescribed dose; V > 107 (%) = volume receiving > 107% but < 110% of the prescribed dose; D2 = dose received by 2% of the target volume; D98 = dose received by 98% of the target volume; D50 = dose received by 50% of the target volume; CI = conformity index; HI = homogeneity index; MU = monitor units; SD = standard deviation.

### Dosimetric parameters for organs at risk

Dosimetric parameters and overdose rates (greater than dose constraints) for OARs in each planning technique are shown in Tables 5 and 6, respectively.

Bone marrow was better protected with VMAT than with C-IMRT (*p* = 0.000) or A-IMRT (*p* = 0.000), whereas both C-IMRT and A-IMRT exhibited similar bone marrow protection (*p* > 0.05). However, the bone marrow dose constraint was exceeded in 4 (20%), 16 (80%), and 18 (90%) patients with VMAT, A-IMRT, and C-IMRT, respectively.

The bladder was better protected with VMAT than with A-IMRT (*p* = 0.000) or C-IMRT (*p* = 0.000). Additionally, the bladder was better protected with A-IMRT than with C-IMRT (*p* = 0.000). Although the bladder dose constraint was exceeded in no patients with A-IMRT or VMAT, it was exceeded in 19 (95%) patients with C-IMRT.

**Table 5:** Dosimetric parameters for organs at risk with each planning technique

	Mean ± SD (minimum–maximum)			Global P
	**C-IMRT**	**A-IMRT**	**VMAT**	
Bone marrow V40 (%)	47.00 ± 6.94^**a**^ (33.21–56.52)	47.79 ± 7.53^**a**^ (33.90–58.12)	35.14 ± 2.33^**b**^ (29.99–40.33)	**0.000** ^*^
Bladder V45 (%)	44.21 ± 6.67^**a**^ (30.15–55.47)	32.84 ± 2.03^**b**^ (28.62–34.88)	25.59 ± 4.72^**c**^ (19.58–34.17)	**0.000** ^*^
Rectum V30 (%)	73.80 ± 4.75^**a**^ (63.64–80.62)	56.18 ± 2.05^**b**^ (52.16–59.77)	49.37 ± 4.82^**c**^ (38.96–58.06)	**0.000** ^*^
Bowel V35 (%)	30.83 ± 9.68^**a**^ (13.41–45.44)	31.27 ± 9.77^**a**^ (14.85–47.57)	27.62 ± 8.17^**b**^ (10.38–39.47)	**0.000** ^*^
Bowel V40 (%)	26.10 ± 8.93^**a**^ (9.11–38.14)	25.76 ± 8.56^**a,b**^ (10.11–39.46)	22.06 ± 6.92^**b**^ (6.51–31.24)	**0.000** ^*^
Right femoral head V30 (%)	21.42 ± 4.03^**a**^ (15.39–27.62)	12.19 ± 1.34^**b**^ (10.33–14.87)	8.71 ± 2.41^**b**^ (4.71–13.30)	**0.000** ^*^
Left femoral head V30 (%)	21.35 ± 4.16^**a**^ (15.20–31.32)	12.58 ± 1.48^**b**^ (9.29–14.85)	8.79 ± 2.39^**c**^ (5.61–14.84)	**0.000** ^*^
Normal tissue (cGy)	1981 ± 423^**a**^ (849–2532)	1966 ± 416^**a**^ (855–2509)	1799 ± 355^**b**^ (785–2235)	**0.000** ^*^

Abbreviations: C-IMRT = 0° angled collimated dynamic IMRT planning technique; A-IMRT = 90° angled collimated dynamic IMRT planning technique; VMAT = volumetric modulated arc therapy; a,b or c = in the comparison between two groups, if the groups have the same letter, there is no statistically significant difference, but if the same letter is not found, there is a statistically significant difference; (*) means statistically significant = p < 0.05; V30 = volume receiving ≥ 30 Gy of the prescribed dose; V35 = volume receiving ≥ 35 Gy of the prescribed dose; V40 = volume receiving ≥ 40 Gy of the prescribed dose; V45 = volume receiving ≥ 45 Gy of the prescribed dose; SD = standard deviation

**Table 6:** Overdose rates (greater than dose constraints) for organs at risk in each planning technique

	C-IMRT *n*(%)	A-IMRT *n*(%)	VMAT *n*(%)	Global P
Bone marrow V40 (%)	18 (90)^**a**^	16 (80)^**a**^	4 (20)^**b**^	**0.000** ^*^
Bladder V45 (%)	19 (95)^**a**^	0 (0)^**b**^	0 (0)^**b**^	**0.000** ^*^
Rectum V30 (%)	20 (100)^**a**^	0 (0)^**b**^	0 (0)^**b**^	**0.000** ^*^
Bowel V35 (%)	9(45)^**a**^	6 (30)^**a**^	4 (20)^**a**^	0.231
Bowel V40 (%)	8 (40)^**a**^	5 (25)^**a**^	4 (20)^**a**^	0.344
Right femoral head V30 (%)	20 (100)^**a**^	0 (0)^**b**^	0 (0)^**b**^	**0.000** ^*^
Left femoral head V30 (%)	20 (100)^**a**^	0 (0)^**b**^	0 (0)^**b**^	**0.000** ^*^

Abbreviations: C-IMRT = 0° angled collimated dynamic IMRT planning technique; A-IMRT = 90° angled collimated dynamic IMRT planning technique; VMAT = volumetric modulated arc therapy; a,b or c = in the comparison between two groups, if the groups have the same letter, there is no statistically significant difference, but if the same letter is not found, there is a statistically significant difference; (*) means statistically significant = p < 0.05; V30 = volume receiving ≥ 30 Gy of the prescribed dose; V35 = volume receiving ≥ 35 Gy of the prescribed dose; V40 = volume receiving ≥ 40 Gy of the prescribed dose; V45 = volume receiving ≥ 45 Gy of the prescribed dose

The rectum was better protected with VMAT than with A-IMRT (*p* = 0.000) or C-IMRT (*p* = 0.000). Additionally, the rectum was better protected with A-IMRT than with C-IMRT (*p* = 0.000). Although the rectum dose constraint was exceeded in no patients with A-IMRT or VMAT, it was exceeded in all (100%) patients with C-IMRT.

The bowel mean V35 (%) value was lower with VMAT than with either dynamic IMRT technique (*p* = 0.000). Additionally, according to the V40 (%) value, the bowel was better protected with VMAT than with C-IMRT (*p* = 0.000). Also, bowel was protected statistically similar with both A-IMRT or C-IMRT (p > 0.05) for both dose constraints. The dose constraint of the bowel (for V40 as in the RTOG 0418 trial) was exceeded in 4 (20%), 5 (25%), 8 (40%) patients with VMAT, A-IMRT, and C-IMRT, respectively.

The left femoral head was better protected with VMAT than with A-IMRT (*p* = 0.000) or C-IMRT (*p* = 0.000). And, it was better protected with A-IMRT than with C-IMRT (*p* = 0.000). Although the right femoral head was protected similar with VMAT or A-IMRT (*p* > 0.05), the lowest protection was observed with C-IMRT (*p* = 0.000). Additionally, whereas dose constraints for both femoral heads were exceeded in no patients with A-IMRT or VMAT, they were exceeded in all (100%) patients with C-IMRT.

NT was exposed to a lower radiation dose with VMAT than with A-IMRT (*p* = 0.000) or C-IMRT (*p* = 0.000). Also, NT exposure dose was similar with C-IMRT and A-IMRT (*p* = 0.05).

## Discussion

RT, one of the main treatment modalities for patients with cancer, is associated with multiple short-term and long-term adverse events. Adverse factors that increase the risk of developing ionizing radiation-induced adverse events in cancer patients are classified into patient-related and treatment-related types. Patient-related adverse factors are primary tumor site, advanced age, female sex, obesity, comorbidities, previous pelvic or abdominal surgery, low body mass index, radiosensitivity-inducing diseases, malnutrition, immune system insufficiency, alcohol drinking, and tobacco smoking. Treatment-related adverse factors are administration of high ionizing radiation dose, large volume of RT, utilization of nonconventional fractionation RT scheme, reirradiation of the same RT field, utilization of different treatment modalities concurrently (e.g., systemic therapy) or sequentially (e.g., brachytherapy), and usage of non-IMRT techniques [21–24].

The incidence of endometrial carcinoma has been increasing because of rising obesity rates and population aging. Endometrial carcinoma is primarily observed in older adults; the median age at diagnosis is between 65 and 76 years. Aging is associated with changes in multiple organs and systems, such as the bone marrow and hematopoietic system, which lead to increased rates of health problems [1, 25, 26]. Bone marrow is the main hematopoietic organ; 51% of its active area is located in the lower spinal and pelvic region [27]. Bone marrow is highly radio- and chemosensitive, and its reserve decreases with age [28]. However, the majority (> 90%) of patients with endometrial carcinoma can undergo surgery [29]. Depending on a patient’s prognostic risk after surgery, pelvic EBRT with/without concurrent systemic therapy and/or vaginal brachytherapy (multimodal treatments) may be necessary. In patients who cannot be treated with brachytherapy (5–10% of patients) [21] or patients with residual metastatic lymph nodes [3, 4], higher doses can be achieved by boosting with EBRT. During the postoperative RT planning process, the small intestine, sigmoid colon, and rectum appear to be displaced toward the target area of RT [5, 7]. Additionally, the life expectancy of patients with endometrial carcinoma is increasing because of advances in cancer diagnosis and treatment. Unfortunately, the risk of recurrence is increased in cancer patients with increased survival, which may result in repeat treatments (e.g., reirradiation of the same region) [30]. Patients with endometrial carcinoma who require treatment with pelvic EBRT have most risk factors that influence the development of adverse events. Therefore, radiation oncologists will encounter cases with both short- and long-term adverse events, which will adversely affect treatment and patient survival [31, 32]. Accordingly, efforts to reduce healthy tissue (or OARs) toxicity are required.

Radiation oncologists should first identify factors associated with possible adverse events, then choose the appropriate treatment modality and irradiation technique; finally, they should inform the patient of necessary precautions and possible adverse events. Factors responsible for the development of adverse events comprise those that can (e.g., IMRT technique) and cannot be changed (e.g., age or previous surgery). The main purpose of RT is to deliver an adequate (or as high as possible) dose to eradicate all cancer cells within the target volume, while minimizing the dose to surrounding healthy tissues [12]. Therefore, the therapeutic ratio will increase with usage, optimization, and development of appropriate RT techniques, thus increasing the rate of successful treatment and decreasing the risk of adverse events.

Adjuvant whole-pelvis EBRT with IMRT/VMAT techniques for endometrial carcinoma is recommended in high–intermediate- and high-risk prognostic groups [3]. The recommended pelvic EBRT dose is between 45 and 50.4 Gy in 25 and 28 fractions, respectively [6, 7, 33]. In this context, if irradiation can be performed only with conventional (collimator angle = 0°) dynamic IMRT, we can meet the dose constraints only when the total dose prescribed to the pelvis is 45 Gy (not 50.4 Gy). As mentioned above, while attempting to increase the whole-pelvis dose to 50.4 Gy, we found that we could deliver the desired dose without exceeding the dose constraints when using a collimator angle of 90° at some gantry angles. Thus, irradiation continued until the initiation of VMAT. When we began VMAT, we wanted to report the 90° angled collimated dynamic IMRT technique, along with a dosimetric comparison to conventional dynamic IMRT and VMAT.

In our dosimetric study, the dynamic IMRT techniques used identical gantry angles to demonstrate the benefit of rotating the collimator angle to 90°. The prescribed pelvic EBRT dose and accepted dose constraints were similar to the methods in the RTOG 0418 [18] and NRG Oncology/RTOG 0123 [7] trials. All plans achieved adequate dose coverage for PTV. Higher mean D2 and V > 107 (%) values were observed with A-IMRT than with either of the other two techniques. However, the maximum detected dose was not > 10% of the prescribed dose (< 5544 cGy) in all planning techniques as in the RTOG 0418 trial. Comparisons between IMRT and VMAT in the literature yielded differences in results with respect to homogeneity, conformality, and NT protection. Homogeneity was reportedly similar for both methods in two studies [13, 34], superior for VMAT in one study [19], and superior for IMRT in one study [35]. Conformality was reportedly similar for both techniques in two studies [19, 35] and superior for VMAT in two studies [13, 34]. NT protection was reportedly similar for both techniques in two studies [13, 19] and superior for VMAT in one study [34]. Although homogeneity was similar for A-IMRT and VMAT in the present study, C-IMRT exhibited the best homogeneity. Whereas conformality was better with C-IMRT than with A-IMRT, the best conformality was observed with VMAT. Although NT protection was similar between A-IMRT and C-IMRT, the best NT protection was observed with VMAT. In C-IMRT, dose constraints were exceeded for bone marrow in 90% of cases, bladder in 95% of cases, rectum in 100% of cases, bowel in 40% of cases (for V40 as in the RTOG 0418 trial), and femoral heads in 100% of cases. We ensured that the gantry angles of the two dynamic IMRT techniques were identical to allow comparisons. We previously mentioned above that even if we used different angles in C-IMRT for a prescribed dose of 50.4 Gy, most of the dose constraints for OARs were exceeded; the present study was conducted as a result of this observation. In the RTOG 0418 trial, a total of 50.4 Gy was applied to the pelvis with the IMRT technique, using the same dose constraints as in the present study. Notably, dose constraints in the RTOG 0418 trial were exceeded for the bladder in 66.7% of cases, rectum in 76.2% of cases, bowel in 16.7% of cases, and femoral heads in 33.3% of cases, even when attempting to plan the optimal treatment with no recommended bone marrow dose constraint and different gantry angles in each patient [6, 18]. In the A-IMRT technique, although bone marrow dose constraints could not be achieved in 80% of cases and bowel in 25% of cases (for V40 as in the RTOG 0418 trial), the dose constraints of the bladder, rectum, and femoral heads were not exceeded in any patient. In this technique, we noted that better dosimetric results were obtained when the appropriate gantry angles (instead of similar gantry angles) were used in each patient. In the VMAT technique, although dose constraints could not be achieved in bone marrow in 20% of cases and bowel in 20% of cases (for V40 as in the RTOG 0418 trial), the dose constraints of the bladder, rectum, and femoral heads were not exceeded in any patient. As a result, OAR protection with A-IMRT was acceptable but inferior to protection with VMAT and superior to protection with C-IMRT. We think that the exposure of OARs to lower ionizing radiation doses with A-IMRT than with C-IMRT is related to the reduction of leakage among multileaf collimators. Finally, the mean MU results between IMRT and VMAT techniques were reportedly lower with VMAT in all previous studies [13, 19, 34, 35]. Rapid irradiation provides additional time for image-guided irradiation, increases patient compliance, and decreases intrafractional patient movement, thus reducing treatment margins and toxicity risk [19]. The main disadvantage of the A-IMRT technique is that it has a higher mean MU than the other two techniques. However, this difference may be overcome with appropriate immobilization tools, considering the risks and benefits for patients.

In conclusion, this study demonstrated that VMAT achieved superior OAR protection with better conformity, compared to A-IMRT and C-IMRT, in patients with endometrial carcinoma. However, VMAT technology is not uniformly available in radiation oncology departments because of its cost. Second, OARs are better protected when EBRT is applied to the pelvis at a dose of 50.4 Gy, by turning the collimator angle to 90° at some gantry angles with the dynamic IMRT technique, when the VMAT technique cannot be performed. This technique may enable better protection of OARs in patients who require ionizing radiation doses > 50.4 Gy (e.g., when brachytherapy cannot be applied or in patients with positive lymph nodes, residual lymph nodes, or tumors) with EBRT. Therefore, further studies are warranted. There is potential for further development of this approach (e.g., its use in the VMAT technique), and its effectiveness in other cancer sites should be investigated.

## Data Availability

The data that support the findings of this study are available from the corresponding author upon reasonable request.

## Abbreviations

AAA: Anisotropic analytical algorithm
A-IMRT: 90° angled collimated dynamic IMRT planning technique
BRA: Brachytherapy
C-IMRT: 0° angled collimated dynamic IMRT planning technique
CI: Conformity index
CT: Computed tomography
CTV: Clinical target volume
DVH: Dose volume histogram
EBRT: External beam radiotherapy
HI: Homogeneity index
ICRU: International Commission on Radiation Units and Measurements
IMRT: Intensity modulated radiotherapy
MU: Monitor unit
NT: Normal tissue
OARs: Organs at risk
PTV: Planning target volume
RT: Radiotherapy
RTOG: Radiation Therapy Oncology Group
TPS: Treatment planning system
VMAT: Volumetric modulated arc therapy
